# Effects of long-term low-dose oxygen supplementation on the epithelial function, collagen metabolism and interstitial fibrogenesis in the guinea pig lung

**DOI:** 10.1186/1465-9921-9-37

**Published:** 2008-04-26

**Authors:** Takuya Aoki, Fumihiro Yamasawa, Takeo Kawashiro, Tetsuichi Shibata, Akitoshi Ishizaka, Tetsuya Urano, Yasumasa Okada

**Affiliations:** 1 Respiratory Division, Department of Internal Medicine, School of Medicine, Tokai University, Isehara, Japan; 2 Marubeni Tokyo Clinic, Marubeni Corporation, Tokyo, Japan; 3 Department of Internal Medicine, Saiseikai Yokohama-City Eastern Hospital, Yokohama, Japan; 4 Pharmaceutical Department, Nihon Pharmaceutical University, Saitama, Japan; 5 Pulmonary Division, Department of Internal Medicine, School of Medicine, Keio University, Tokyo, Japan; 6 Department of Medicine, Tsukigase Rehabilitation Center, Izu, Japan

## Abstract

**Background:**

The patient population receiving long-term oxygen therapy has increased with the rising morbidity of COPD. Although high-dose oxygen induces pulmonary edema and interstitial fibrosis, potential lung injury caused by long-term exposure to low-dose oxygen has not been fully analyzed. This study was designed to clarify the effects of long-term low-dose oxygen inhalation on pulmonary epithelial function, edema formation, collagen metabolism, and alveolar fibrosis.

**Methods:**

Guinea pigs (n = 159) were exposed to either 21% or 40% oxygen for a maximum of 16 weeks, and to 90% oxygen for a maximum of 120 hours. Clearance of inhaled technetium-labeled diethylene triamine pentaacetate (Tc-DTPA) and bronchoalveolar lavage fluid-to-serum ratio (BAL/Serum) of albumin (ALB) were used as markers of epithelial permeability. Lung wet-to-dry weight ratio (W/D) was measured to evaluate pulmonary edema, and types I and III collagenolytic activities and hydroxyproline content in the lung were analyzed as indices of collagen metabolism. Pulmonary fibrotic state was evaluated by histological quantification of fibrous tissue area stained with aniline blue.

**Results:**

The clearance of Tc-DTPA was higher with 2 week exposure to 40% oxygen, while BAL/Serum Alb and W/D did not differ between the 40% and 21% groups. In the 40% oxygen group, type I collagenolytic activities at 2 and 4 weeks and type III collagenolytic activity at 2 weeks were increased. Hydroxyproline and fibrous tissue area were also increased at 2 weeks. No discernible injury was histologically observed in the 40% group, while progressive alveolar damage was observed in the 90% group.

**Conclusion:**

These results indicate that epithelial function is damaged, collagen metabolism is affected, and both breakdown of collagen fibrils and fibrogenesis are transiently induced even with low-dose 40% oxygen exposure. However, these changes are successfully compensated even with continuous exposure to low-dose oxygen. We conclude that long-term low-dose oxygen exposure does not significantly induce permanent lung injury in guinea pigs.

## Background

Chronic obstructive pulmonary disease (COPD) has been expected to become a major cause of morbidity and mortality worldwide [1-3]. The patient population receiving long-term oxygen therapy has been increasing with rising morbidity of COPD. Although oxygen supplementation is indispensable in the management of hypoxemia in patients with various respiratory disorders such as COPD, high-dose oxygen inevitably produces free radicals [4]. High-dose oxygen also induces lethal pulmonary injury in humans as well as various animal models [5-10]. However, the potential lung injury caused by long-term exposure to relatively low-dose supplemented oxygen has not been fully analyzed. Exposure to low-dose oxygen (less than 50%) has been reported to induce no designable injury [5,7]. On the other hand, there have also been contradicting reports; an increase of albumin concentration in human bronchoalveolar lavage (BAL) fluid with only 30% oxygen exposure for 45 hours, alterations in type II pneumocytes with relatively low (60%) oxygen exposure in rats [11], and increased lung catalase activity with 50% oxygen exposure in rats [12]. Exposure to 60% oxygen for 2 weeks was recently demonstrated to induce thickening of inter-alveolar septa, intense cellular infiltration and deposition of interstitial collagen fibers in rats [13].

The discrepant results regarding the pulmonary toxicity of relatively low-dose oxygen exposure may be attributed to the different injury markers, oxygen concentrations and exposure durations used in these studies. In order to elucidate the mechanism of possible lung injuries induced by chronic exposure to an adaptive dose of oxygen, selections of systemic injury markers, the oxygen concentrations used and exposure duration are critical. Therefore, we aimed to clarify the effects of long-term low-dose oxygen inhalation on lung epithelial permeability, induction of pulmonary edema, collagen metabolism and interstitial fibrosis by exposing guinea pigs to various oxygen concentrations.

## Methods

### Animals and oxygen exposure

Specific pathogen-free male Hartley guinea pigs (Sankyo Labo Service, Tokyo, Japan), 4 weeks of age at the beginning of exposure, weighing 270–300 g, were used (n = 159). All of the experimental protocols were approved by the institutional Animal Experiment Committee and conformed to the Guide for the Care and Use of Laboratory Animals published by the National Institutes of Health.

Oxygen exposure was conducted by placing animals in a semi-sealed vinyl isolation chamber (volume 450 L), throughout the experimental period. Sterile food and water were provided regularly through a double outlet, and the chamber was kept clean with daily care. Through a 0.2 micrometer filter, 40% oxygen or room air was delivered at a flow rate of 15 L/min. Oxygen and carbon dioxide concentrations in the chamber were measured with a gas analyzer (Type 1312, Instrumentation Laboratory, Lexington, MA). The oxygen concentration was maintained and consistently confirmed to be 21 ± 1% (mean ± SD), 40 ± 2% or 90 ± 3%, and the carbon dioxide concentration was always below 0.5% at the outlet of the chamber. Humidity was maintained at 55–60%.

Total animal numbers for the examination of BAL/Serum ALB and TP, lung W/D or collagen metabolism were n = 10 in the pre-exposure group; n = 8 (2 weeks), 10 (4 weeks), 6 (8 weeks) and 6 (16 weeks) in the 40% oxygen exposure groups; n = 5 (2 weeks), 8 (4 weeks), 6 (8 weeks) and 6 (16 weeks) in the 21% oxygen exposure groups; n = 5 (24 hours), 5 (48 hours), 5 (72 hours), 10 (96 hours) and 5 (120 hours) in the 90% oxygen exposure groups. For examination of the clearance of inhaled Tc-DTPA: n = 5 in the pre-exposure group; n = 5 in each (2, 4, 8 or 16 weeks) 40% oxygen exposure group; n = 5 in each (2, 4, 8 or 16 weeks) 21% oxygen exposure group; n = 5 (24 hours), 6 (48 hours) and 8 (72 hours) in the 90% oxygen exposure groups.

### Assay of inhaled Tc-DTPA clearance

Sixty millicuries of ^99 m^TcO_4_^- ^were eluted from a ^99 m^Mb-^99 m^Tc generator and bound to DTPA (Daiichi Radioisotope Labs, Tokyo). Eight milliliters of Tc-DTPA saline solution were aerosolized using an ultrasonic nebulizer (Model 65, Devilbiss Sunrise Medical, Carlsbad, CA). The aerosol was kept in the bag for 5 min to allow the large droplets to settle to the bottom [14]. The animals were placed supine, and Tc-DTPA aerosol was delivered via the cannulated inspiratory circuit under spontaneous breathing. Radioactivity was measured each minute for 10 minutes in the anterior view using a gamma camera (GCA 401-5, Toshiba, Tokyo). A logarithmic plot of activity vs time was obtained from a region of interest. We corrected for physical decay and the background was subtracted. Monoexponential equations were fitted to the curves by the least squares method and the clearance rate was expressed as kep.

### Histological examination

Deep surgical anesthesia was achieved with 100 mg/kg of ketamine (Ketalar; Parke Davis & Co., Detroit, MI) and 5 mg/kg of xylazine (Rompun; Cutter Laboratories, Shawnee, KS) injected intraperitonealy. The animals were then sacrificed by exsanguination. The chest was opened, and the lung was taken out of the thorax. A catheter was inserted into the right bronchus. The right lung tissue was embedded in paraffin after fixation with 4% paraformaldehyde in 0.1 M phosphate buffer (pH 7.4) for 24 hours at 4°C. Three sections (each 3 μm thick) were made from the upper, middle and lower lobes at an equal distance from the top to the bottom of the right lung and stained with aniline blue or hematoxylin-eosin.

### Determination of fibrous tissue stained with aniline blue

**Figure 1:**
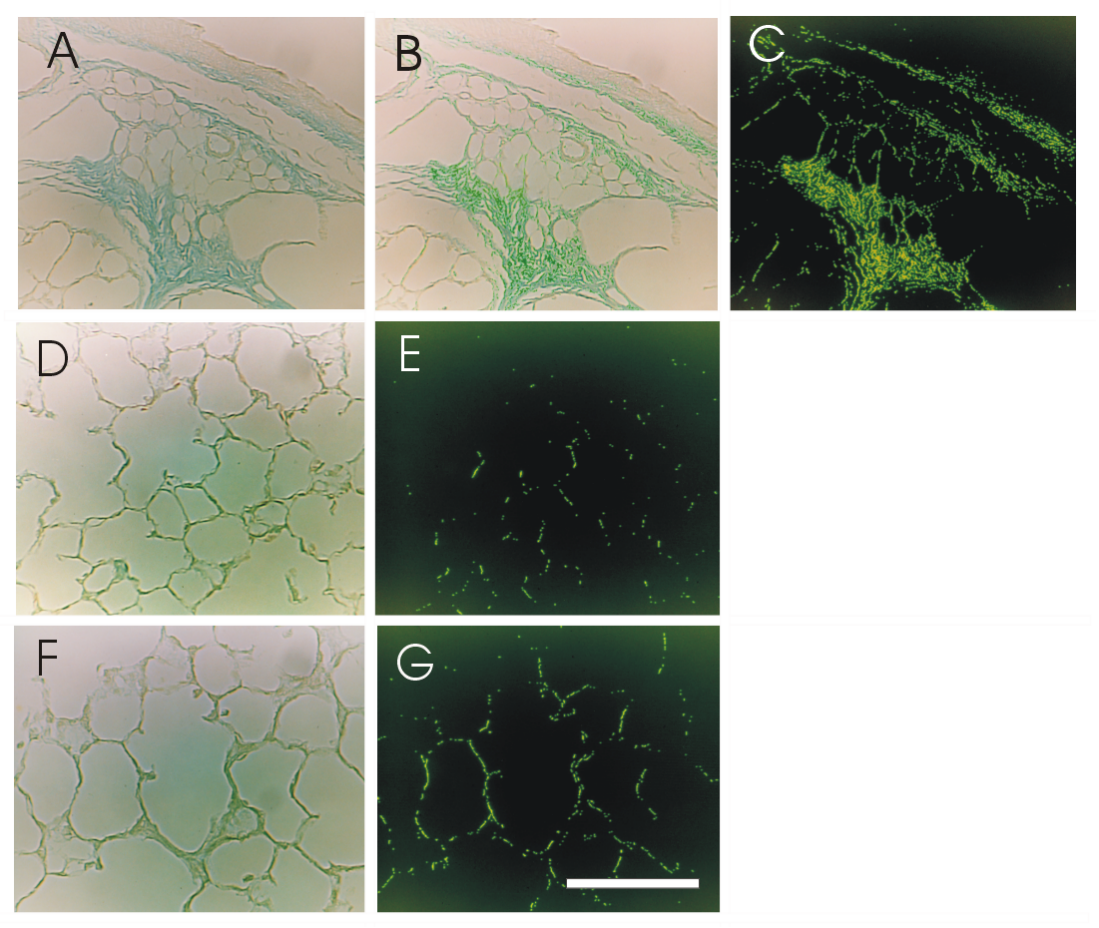
**Determination of fibrous tissue stained with aniline blue and peripheral lung sections with 2 week exposure to room air or 40% oxygen**. The area of fibrous tissue stained with aniline blue (A) was selected with the color image analyzing system (B). The stained portion and the extracted portion were matched. The selected area was measured and expressed as a percentage of the total area (C). Panels D and F show staining with aniline blue. Panels E and G show areas of fibrous tissue selected with the image analyzing system. Panels D and E are from animals on room air, F and G from those on 40% oxygen, after 2 weeks of exposure. Bar = 100 μm.

The area of aniline blue-stained fibrous tissue, which mainly consists of collagen fibers, was measured with a color image analyzing system (SP500, Olympus, Tokyo). An area of fibrous tissue around a bronchus stained with aniline blue (Fig. 1A) was selected with the color image analyzing system (Fig. 1B). The stained portion and the extracted portion were matched. The selected area was measured and expressed as a percentage of the total area (Fig. 1C) [15]. The fibrous tissue area was measured using ten different fields of three different sections corresponding to the upper, middle and lower lobes of the right lung. These areas were selected at random from peripheral parts of the lung. If there was a bronchus, a large vessel or pleura inside the area, another area was selected because collagen fibers were abundant around these tissues. The fibrous tissue area to total area was expressed as the mean ± SD. Histological changes in hematoxylin-eosin stained lung sections with 0, 2, 4, 8 and 16 week-exposure periods in the 40% oxygen groups and pre, 72, 96 and 120 hour-exposure periods in the 90% oxygen groups were also examined.

### Lung water measurement

The lung W/D in the left caudal lobe was measured to quantify the pulmonary edema induced by 40% or 90% oxygen exposure. After the wet weights of the lung tissue samples had been measured, the tissues were completely dried in a vacuum oven (DP22; Yamato Scientific, Tokyo) at 95°C and at 0 cmH_2_O for 48 hours [16].

### Bronchoalveolar lavage

Bronchoalveolar infiltrating cells were assessed by BAL of the left cranial lobe [16]. The lobe was weighed and its bronchus was catheterized, then lavaged twice with 5 ml of saline and 1 ml of air. BAL fluid was collected, placed on slides, then fixed and stained with a modified Wright's staining technique (Diff-Quik; American Scientific Products, McGraw Park, IL). BAL cell differentials were based on the means of five separate counts of 100 cells on the high power field of light microscopic slides. BAL fluid samples were centrifuged at 400 × g at 4°C for 10 min to obtain a cell pellet. Each cell pellet was suspended in 1 ml of saline, and a cell count was obtained using a modified hematocytometer method (Unopet Microcollection System; Becton Dickinson, Rutherford, NJ). BAL cell counts were expressed as counts per 1 mg lung tissue. The albumin concentrations in plasma and BAL fluid were determined using the bromcresol green dye binding method (Sigma Chemical Co., St. Louis, MO) [17]. Total protein was determined using the Biuret method. The BAL fluid-to-serum (BAL/Serum) albumin (ALB) and total protein (TP) ratios were used as parameters of alveolar-capillary permeability [18] and lung injury [19], respectively.

### Hydroxyproline measurement

Measurement of total collagen contents in the peripheral portions of the lung was based on the estimation of hydroxyproline contents. Saline solution (1.5 ml) was added to an approximately 30 mg tissue sample which was collected from the peripheral part of the left lower lobe. Then, the sample was homogenized on ice and used for the hydroxyproline content measurement, and to determine types I and III collagenolytic enzyme activities. Hydroxyproline contents were measured by the spectrophotometrical method [20,21], which allows quantification of the amount of hydroxyproline in a small sample.

### Purification and labeling of type III collagen

Pure type III collagen was prepared according to the method of Glanville [22], and type III collagen labeling was performed as previously described [23].

### Assays of collagenolytic enzyme activities

In order to measure type I and type III collagenolytic activities in lung tissues, we utilized bacterial collagenase to obtain a standard curve [23]. Purified collagenase from *Clostridium histolyticum *(EC 3.4.24.3, bacterial collagenase type II, Sigma Chemical, St. Louis, MO) was dissolved in Tris-HCl buffer to obtain a 1 mg/ml concentration of the standard solution, which was then diluted to various concentrations (10^-1 ^to 10^-5 ^mg/ml) to provide the standard curves of types I and III collagenolytic enzyme activities. One unit of collagenolytic enzyme activity was defined as the weight of the bacterial collagenase having the same collagenolytic activity (μg bacterial collagenase/g protein) as the lung tissue sample. This standardization with bacterial collagenase made it possible to compare types I and III collagenolytic activities [23].

### Protein contents

Protein contents of the samples were determined by the Lowry method [24]. Hydroxyproline content was expressed as μg per 1 mg protein, and types I and III collagenolytic activities were expressed as μg bacterial collagenase per 1 g protein.

### Statistical analysis

The results were presented as means ± standard deviation (SD). Statistical analyses were conducted using one-way analysis of variance (ANOVA) followed by Tukey post-hoc test to detect differences among groups. Differences between two groups were assessed using the unpaired t-test or Welch's test. A p-value < 0.05 was considered statistically significant.

## Results

### Changes in BAL cells, BAL/Serum Alb and TP ratio

**Figure 2:**
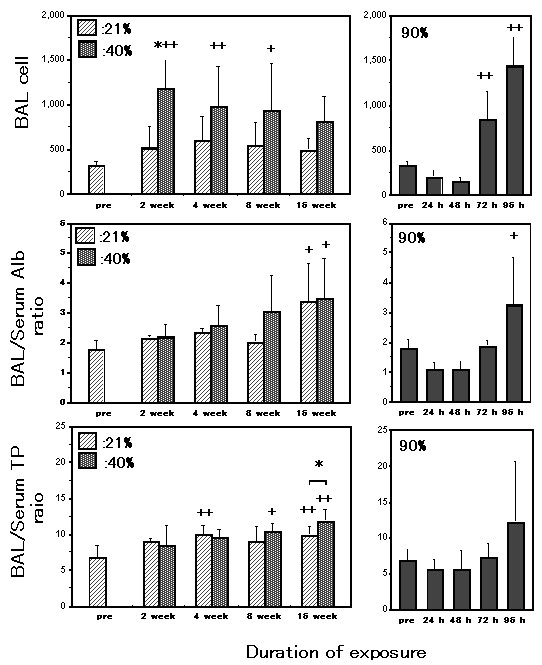
**Changes in BAL cells, BAL/Serum Alb and TP ratio**. Broncho-alveolar infiltrating cells (top panel) were expressed as × 10^2 ^counts/mg lung tissue. The average count of BAL cells was higher in the 40% oxygen group than in the 21% oxygen group. The increase was statistically significant after 2 weeks of exposure. BAL cell counts in the 40% oxygen 2 week-, 4 week- and 8 week-exposure group were significantly increased as compared to those in the pre-exposure group. After 72 hour exposure to 90% oxygen, BAL cell counts were increased as compared to the pre-exposure level. n = 9 in the pre-exposure group; 8 (2 weeks), 9 (4 weeks), 6 (8 weeks) and 5 (16 weeks) in the 40% oxygen exposure groups; n = 4 (2 weeks), 8 (4 weeks), 5 (8 weeks) and 5 (16 weeks) in the 21% oxygen exposure groups; n = 5 (24 hours), 4 (48 hours), 5 (72 hours) and 8 (96 hours) in the 90% oxygen exposure groups. The BAL/Serum Alb ratios (middle panel) in the 40% oxygen groups did not differ from those in the 21% exposure groups at any of the durations examined. The Alb ratio in the 40% or 21% oxygen 16 week-exposure group was significantly increased as compared to those in the pre-exposure group. This increase, however, did not differ between the 40% and the 21% oxygen groups. After 96 hours of exposure to 90% oxygen, the Alb ratio was increased as compared to the pre-exposure level. n = 5 in the pre-exposure group; 8 (2 weeks), 9 (4 weeks), 6 (8 weeks) and 6 (16 weeks) in the 40% oxygen exposure groups; 6 (2 weeks), 7 (4 weeks), 5 (8 weeks) and 6 (16 weeks) in the 21% oxygen exposure groups; 5 (24 hours), 5 (48 hours), 5 (72 hours) and 6 (96 hours) in the 90% oxygen exposure groups. The BAL/Serum TP ratios (bottom panel) in the 40% oxygen groups did not differ from those in the 21% exposure groups at 2, 4 and 8 week-exposure periods. After 16 weeks of exposure, the BAL/Serum TP ratio in the 40% oxygen group was higher than that in the 21% 16 week-exposure group. The BAL/Serum TP ratio in the 40% oxygen 8 and 16 week-exposure groups and the 21% oxygen 4 and 16 week-exposure groups were significantly increased as compared to those in the pre-exposure group. After 96 hours of exposure to 90% oxygen, the increased TP ratio was not statistically significant as compared to the pre-exposure level. n = 5 in the pre-exposure group; 8 (2 weeks), 7 (4 weeks), 6 (8 weeks) and 5 (16 weeks) in the 40% oxygen exposure groups; 6 (2 weeks), 8 (4 weeks), 5 (8 weeks) and 6 (16 weeks) in the 21% oxygen exposure groups; 5 (24 hours), 5 (48 hours), 5 (72 hours) and 5 (96 hours) in the 90% oxygen exposure groups. Values are expressed as means ± SD; * p < 0.05 as compared to the value of the 21% oxygen exposure duration-matched control. + p < 0.05 and ^++ ^p < 0.01 as compared to the value of the pre-exposure group.

The average count of BAL cells was higher in the 40% than in the 21% oxygen groups, and this increase was statistically significant after 2 weeks of exposure (Fig. 2). BAL cell counts in the 40% oxygen 2 week-, 4 week- and 8 week-exposure groups were significantly increased as compared to those in the pre-exposure group. After 72 hour exposure to 90% oxygen, BAL cell counts were increased as compared to the pre-exposure level. The percentages of the BAL neutrophils were not different between the 40% and the 21% oxygen groups at any time examined (Table 1). The percentages of the BAL macrophages and lymphocytes were not different between the 40% and the 21% oxygen groups either (Table 1). On the other hand, after 24 and 48 hour exposure to 90% oxygen, the percentages of the BAL eosinophils were significantly increased as compared to those in the pre-exposure condition (Table 2). After 72 hour exposure to 90% oxygen, the percentage of the BAL neutrophils was significantly increased as compared to the pre-exposure level (Table 2).

**Table 1:** Changes in nucleated cells in BAL fluid during 40% oxygen exposure

		**pre**	**2 weeks**	**4 weeks**	**8 weeks**	**16 weeks**
**Macrophage (%)**	**40% oxygen**		86.9 ± 4.0	88.8 ± 4.6	91.5 ± 3.4	92.8 ± 1.9
	**Room air**	91.1 ± 2.3	89.8 ± 3.7	87.5 ± 5.0	92.4 ± 1.5	89.6 ± 3.6
**Lymphocyte (%)**	**40% oxygen**		12.1 ± 3.8	10.0 ± 4.6	7.0 ± 3.0	6.4 ± 1.9
	**Room air**	8.1 ± 2.6	8.5 ± 2.4	10.9 ± 5.2	5.8 ± 0.8	9.2 ± 4.0
**Neutrophil (%)**	**40% oxygen**		1.1 ± 1.0	1.2 ± 0.8	1.5 ± 2.1	0.8 ± 0.8
	**Room air**	0.8 ± 1.0	1.8 ± 2.1	1.5 ± 1.1	1.8 ± 0.8	1.2 ± 1.1

Values are mean ± SD.

**Table 2:** Changes in nucleated cells in BAL fluid during 90% oxygen exposure

	**pre**	**24 hours**	**48 hours**	**72 hours**	**96 hours**	**120 hours**
**Macrophage (%)**	90.8 ± 3.8	85.6 ± 2.6	80.7 ± 3.2^++^	85.0 ± 3.3^+^	73.1 ± 5.7^++^	53.7 ± 7.7^++^
**Lymphocyte (%)**	8.3 ± 4.0	8.8 ± 0.8	16.0 ± 3.6^++^	4.0 ± 1.2^+^	7.5 ± 3.1	5.8 ± 1.7
**Neutrophil (%)**	0.8 ± 0.8	1.0 ± 2.2	0.0 ± 0.0	11.0 ± 2.7^++^	18.5 ± 7.0^++^	40.3 ± 7.8^++^
**Eosinophil (%)**	0.0 ± 0.0	4.6 ± 1.1^++^	3.3 ± 0.4^++^	0.0 ± 0.0	0.0 ± 0.0	0.0 ± 0.0

Values are mean ± SD. + p < 0.05, ++ p < 0.01 as compared to the pre-exposure level.

The BAL/Serum Alb ratio in the 40% oxygen groups did not differ from that in the 21% exposure groups for any of the durations examined (Fig. 2). The Alb ratio in the 40% or 21% oxygen 16 week-exposure group was significantly increased as compared to those in the pre-exposure group. This increase, however, did not differ between the 40% and the 21% oxygen groups. After 96 hour exposure to 90% oxygen, the Alb ratio was significantly increased as compared to the pre-exposure level.

The BAL/Serum TP ratio in the 40% oxygen groups did not differ from that in the 21% exposure groups with 2, 4 and 8 week exposures (Fig. 2). After 16 weeks of exposure, the BAL/Serum TP ratio in the 40% oxygen group was higher than that in the 21% exposure group. The BAL/Serum TP ratio in the 40% oxygen 8 and 16 week-exposure groups and the 21% oxygen 4 and 16 week-exposure groups were significantly increased as compared to those in the pre-exposure group. After 96 hours of exposure to 90% oxygen, the increased TP ratio was not statistically significant as compared to the pre-exposure level.

### Lung clearance of inhaled Tc-DTPA

**Figure 3:**
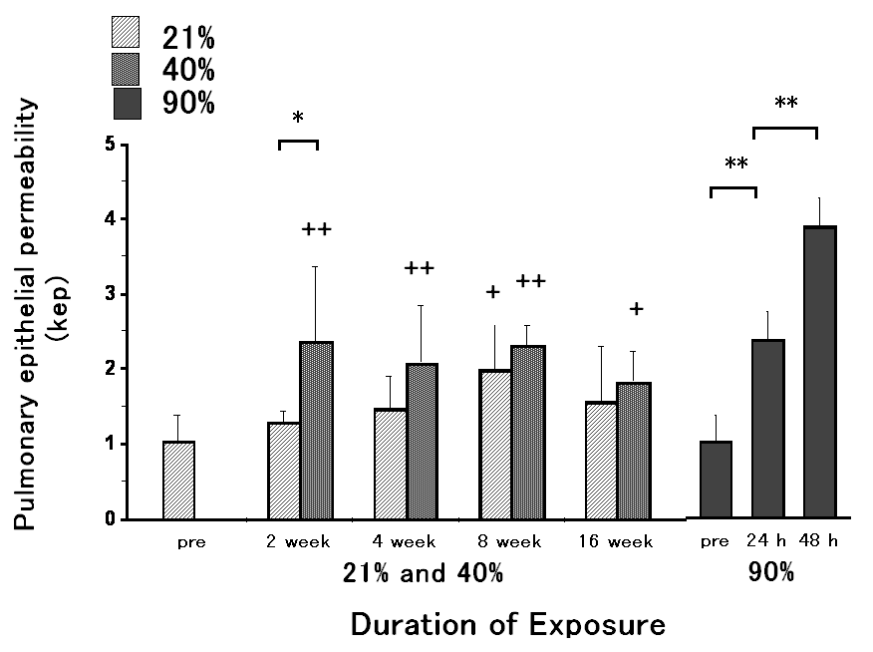
**Lung clearance of inhaled Tc-DTPA**. The kep of the 40% oxygen 2 week-exposure group was increased than that of the 21% oxygen 2 week-exposure group. The kep of the 40% oxygen 2, 4, 8 and 16 week-exposure groups and the 21% oxygen 8 week-exposure group were significantly increased as compared to that of the pre-exposure group. This increase, however, did not differ between the 40% and the 21% oxygen groups. The kep of the 90% oxygen 24 hour-exposure group was higher than that of the pre-exposure group, and that of the 48 hour-exposure group was higher than that of the 24 hour-exposure group. Values are expressed as means ± SD; * p < 0.05 and ** p < 0.01 as compared to the value of the 21% oxygen exposure duration-matched control or pre and the 90% oxygen 24 hour-exposure group. ^+ ^p < 0.05 and ^++ ^p < 0.01 as compared to the value of the pre-exposure group. n = 5 in the pre-exposure group; n = 5 in each 40% oxygen exposure group; n = 5 in each 21% oxygen exposure group; n = 5 (24 hours), 6 (48 hours) and 8 (72 hours) in the 90% oxygen exposure groups.

The kep of the 40% oxygen 2 week-exposure group was higher than that of the 21% oxygen 2 week-exposure group (Fig. 3). The kep of the 40% oxygen 2, 4, 8 and 16 week-exposure groups and the 21% oxygen 8 week-exposure group were significantly increased as compared to that of the pre-exposure group. This increase did not differ between the 40% and the 21% oxygen groups. The kep of the 90% oxygen 24 hour-exposure group was higher than that of the pre-exposure group, and that of the 48 hour-exposure group was higher than that of the 24 hour group.

### Lung W/D

**Figure 4:**
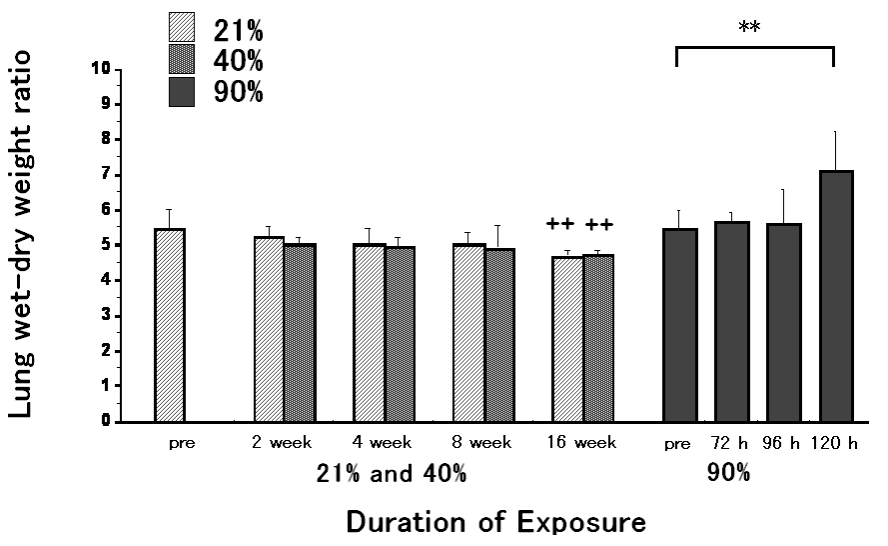
**Lung W/D as a marker of pulmonary edema**. No significant differences were observed between the 40% and 21% oxygen groups at any of the durations examined. The lung W/D of the 40% or 21% oxygen 16 week-exposure group was significantly decreased as compared to that of the pre-exposure group. Lung W/D of the 120 hour-exposure group was increased as compared to that of the pre-exposure group. Values are expressed as means ± SD; ** p < 0.01 and ^++ ^p < 0.01 as compared to the value of the pre-exposure group. n = 8 in the pre-exposure group; n = 8 (2 weeks), 10 (4 weeks), 5 (8 weeks) and 6 (16 weeks) in the 40% oxygen exposure groups; n = 5 (2 weeks), 8 (4 weeks), 6 (8 weeks) and 6 (16 weeks) in the 21% oxygen exposure groups; n = 5 (72 hours), 5 (96 hours) and 5 (120 hours) in the 90% oxygen exposure groups.

No significant differences were observed between the 40% and 21% oxygen groups at any of the durations examined (Fig. 4). The lung W/D of the 40% or 21% oxygen 16 week-exposure group was significantly decreased as compared to that of the pre-exposure group. Lung W/D of the 120 hour-exposure group was increased as compared to that of the pre-exposure group.

### Type I collagenolytic enzyme activity

**Figure 5:**
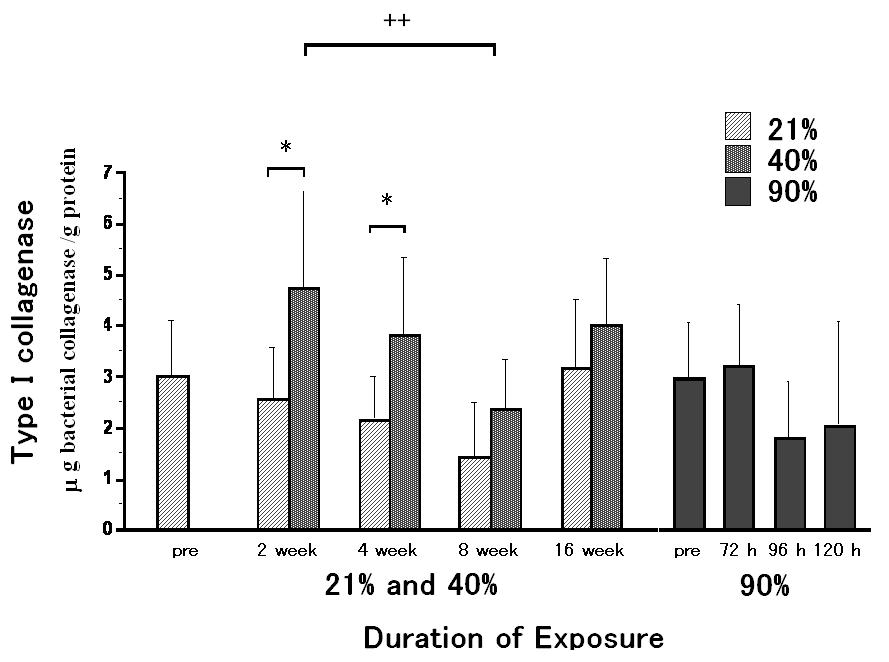
**Type I collagenolytic enzyme activity**. Type I collagenolytic enzyme activity in the 40% oxygen 2 week-exposure group was significantly higher than that in the 21% oxygen 2 week-exposure group (Fig. 5). This increase was, however, not significantly different as compared to that in the pre-exposure group. Type I collagenolytic activity in the 40% oxygen 4 week-exposure group was also higher than that in the 21% oxygen 4 week-exposure group, although not significantly higher than that before exposure. Type I collagenolytic activity in the 40% oxygen 8 week-exposure group was lower than that in the 40% oxygen 2 week-exposure group. After 8 and 16 weeks of exposure, there were no differences between the 40% and 21% exposure groups or between the 40% and pre-exposure groups. There were no significant differences among the 90% oxygen exposure groups. Values are expressed as means ± SD; n = 9 in the pre-exposure group; n = 8 (2 weeks), 10 (4 weeks), 6 (8 weeks) and 6 (16 weeks) in the 40% oxygen exposure groups; n = 5 (2 weeks), 8 (4 weeks), 6 (8 weeks) and 6 (16 weeks) in the 21% oxygen exposure groups; n = 4 (72 hours), 5 (96 hours) and 5 (120 hours) in the 90% oxygen exposure groups. * p < 0.05 as compared to the value of the 21% oxygen exposure duration-matched control. ^++ ^p < 0.01 as compared to the value of the 40% oxygen 2 week-exposure group.

Type I collagenolytic enzyme activity in the 40% oxygen 2 week-exposure group was significantly higher than that in the 21% oxygen 2 week-exposure group (Fig. 5). This increase was, however, not significantly different as compared to that in the pre-exposure group. Type I collagenolytic activity in the 40% oxygen 4 week-exposure group was also higher than that in the 21% oxygen 4 week-exposure group, although not significantly higher than that before exposure. Type I collagenolytic activity in the 40% oxygen 8 week-exposure group was lower than that in the 40% oxygen 2 week-exposure group. After 8 or 16 weeks of exposure, there were no differences between the 40% and 21% exposure groups or between the 40% and pre-exposure groups. There were no significant differences among the 90% oxygen exposure groups.

### Type III collagenolytic enzyme activity

**Figure 6:**
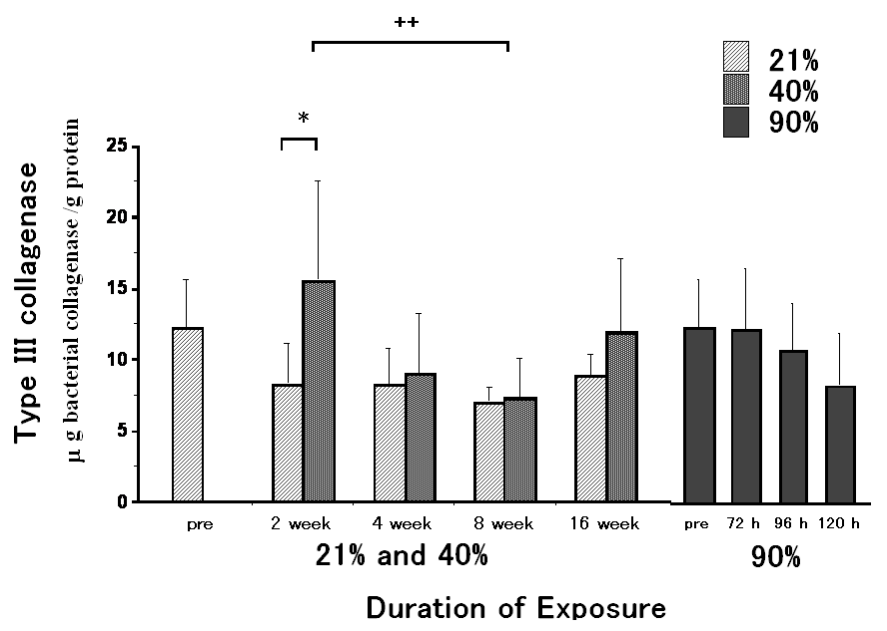
**Type III collagenolytic enzyme activity**. Type III collagenolytic enzyme activity in the 40% oxygen 2 week-exposure group was significantly increased as compared to that in the 21% oxygen 2 week-exposure group. This increase was, however, not significantly different as compared to that in the pre-exposure group. At 4, 8 and 16 week-exposure periods, there were no differences in type III collagenolytic enzyme activity between the 40% and the 21% exposure groups, or between the 40% and the pre-exposure groups. Type III collagenolytic activities in the 40% oxygen 8 week-exposure groups was decreased as compared to that in the 40% oxygen 2 week-exposure group. There was no significant difference among the 90% oxygen exposure groups. Values are expressed as means ± SD; n = 8 in the pre-exposure group; n = 7 (2 weeks), 10 (4 weeks), 6 (8 weeks) and 6 (16 weeks) in the 40% oxygen exposure groups; n = 5 (2 weeks), 8 (4 weeks), 5 (8 weeks) and 6 (16 weeks) in the 21% oxygen exposure groups; n = 5 (72 hours), 5 (96 hours) and 5 (120 hours) in the 90% oxygen exposure groups. * p < 0.05 as compared to the value of the 21% oxygen exposure duration-matched control. ++ p < 0.01 as compared to the value of the 40% oxygen 2 week-exposure group.

Type III collagenolytic enzyme activity in the 40% oxygen 2 week-exposure group was significantly higher than that in the 21% oxygen 2 week-exposure group (Fig. 6). This increase was, however, not significantly different as compared to that in the pre-exposure group. After 4, 8 or 16 weeks of exposure, there were no differences in type III collagenolytic enzyme activity between the 40% and 21% exposure groups, or between the 40% and pre-exposure groups. Type III collagenolytic activities in the 40% oxygen 8 week-exposure group was lower than that in the 40% oxygen 2 week-exposure group. There were no significant differences among the 90% oxygen exposure groups.

### Changes in hydroxyproline content during oxygen exposure

**Figure 7:**
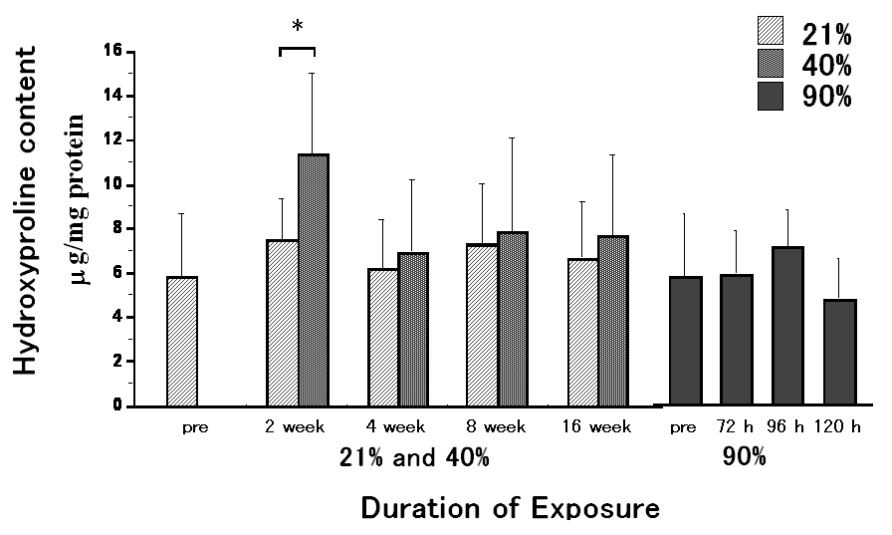
**Hydroxyproline content during oxygen exposure**. The hydroxyproline content with 2 week exposure to 40% oxygen was significantly increased as compared to that with 2 week exposure to 21% oxygen. This increase was not significantly different as compared to that in the pre-exposure group. At 4, 8 and 16 week-exposure periods, there were no differences in hydroxyproline content between the 40% and the 21% exposure groups, or between the 40% and the pre-exposure groups. There were no significant differences among the 90% oxygen exposure groups. Values are expressed as means ± SD; n = 9 in the pre-exposure group; n = 5 (2 weeks), 9 (4 weeks), 5 (8 weeks) and 6 (16 weeks) in the 40% oxygen exposure groups; n = 5 (2 weeks), 8 (4 weeks), 5 (8 weeks) and 5 (16 weeks) in the 21% oxygen exposure groups; n = 5 (72 hours), 5 (96 hours) and 5 (120 hours) in the 90% oxygen exposure groups. * p < 0.05 as compared to the value of the 21% oxygen exposure duration-matched control.

The hydroxyproline content with 2 week exposure to 40% oxygen was significantly increased as compared to that with 2 week exposure to 21% oxygen (Fig. 7). This increase was not significantly different as compared to that in the pre-exposure group. After 4, 8 or 16 weeks of exposure, there were no differences in hydroxyproline content between the 40% and 21% exposure groups, or between the 40% and pre-exposure groups. There were no significant differences among the 90% oxygen exposure groups.

### Quantification of fibrous tissue area

**Figure 8:**
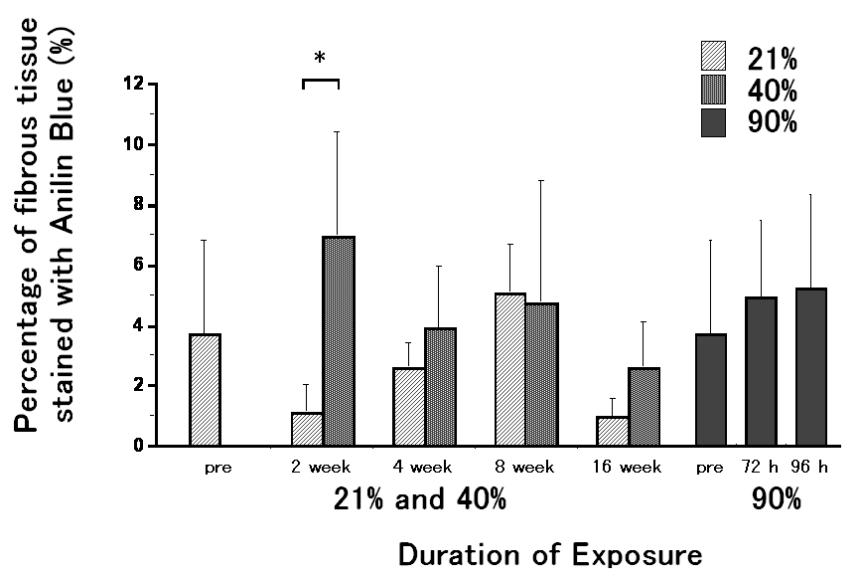
**Percentage of fibrous tissue stained with aniline blue**. The percentage of fibrous tissue with 2 week exposure to 40% oxygen was significantly increased as compared to that with 2 week exposure to 21% oxygen. There was no significant difference among the 90% oxygen exposure groups. Fibrous tissue levels in 2 and 16 week 21% oxygen exposure control lungs were not lower as compared to that in pre-exposure. Values are expressed as means ± SD. n = 10 in the pre-exposure group; n = 8 (2 weeks), 10 (4 weeks), 6 (8 weeks) and 6 (16 weeks) in the 40% oxygen exposure groups; n = 4 (2 weeks), 7 (4 weeks), 6 (8 weeks) and 5 (16 weeks) in the 21% oxygen exposure groups; n = 5 (72 hours) and 9 (96 hours) in the 90% oxygen exposure groups. * p < 0.01 as compared to the value of the 21% exposure duration-matched control.

Sections of peripheral parts of the lungs stained with aniline blue and the estimated area of fibrous tissue with 2 week exposure to 21% or 40% oxygen are shown in Fig. 1. The percentage of fibrous tissue with 2 week exposure to 40% oxygen was significantly increased as compared to that with 2 week exposure to 21% oxygen (Fig. 8). There were no differences between the 40% and 21% exposure groups at 4, 8 or 16 weeks. There were no significant differences among the 90% oxygen exposure groups. Fibrous tissue levels in 2 and 16 week-21% oxygen groups were not lower as compared to that in pre-exposure

### Sections of peripheral parts of the lungs stained with hematoxylin-eosin

**Figure 9:**
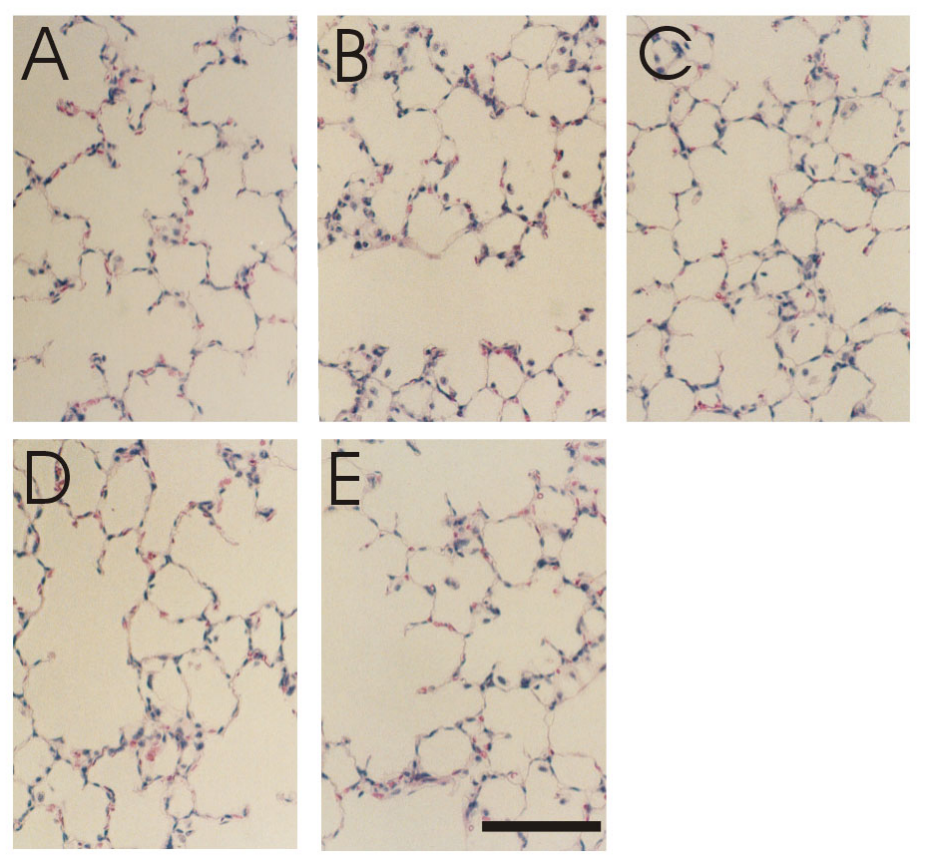
**Sections of peripheral parts of the lungs stained with hematoxylin-eosin**. Panels A, B, C, D and E represent pre- and, 2, 4, 8 and 16 week-exposure to 40% oxygen. Neither discernible injury nor architectural disorganization can be seen in any of these groups. Bar = 100 μm.

**Figure 10:**
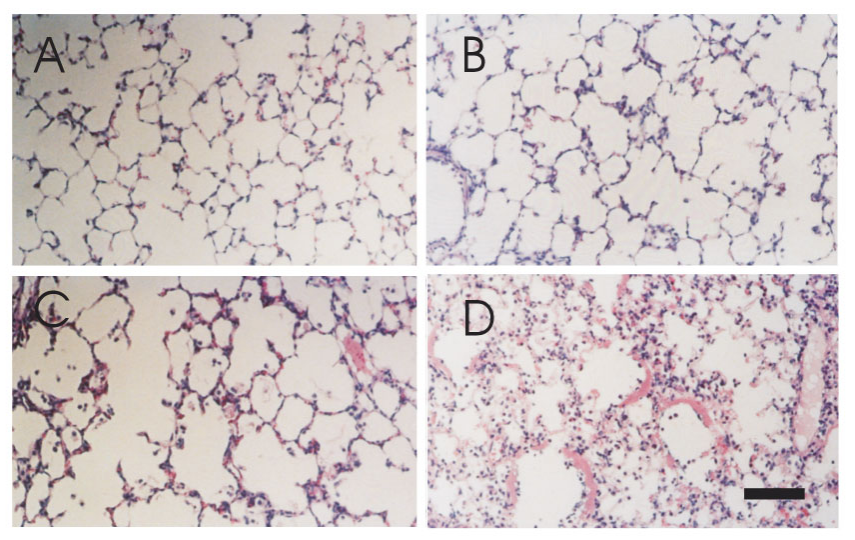
**Sections of peripheral parts of the lungs stained with hematoxylin-eosin**. Panels A, B, C and D represent pre- and, 72, 96 and 120 hour-exposure to 90% oxygen. Inflammatory cell infiltration was recognized at 72 hours of exposure, and alveolar septal destruction was apparent by 96 hours of exposure. Diffuse alveolar damage was apparent by 120 hours of exposure. Bar = 100 μm.

Although the image analyzing system revealed that the fibrous tissue in the aniline blue stained section was increased with 2 weeks exposure to 40% oxygen, neither discernible injury nor architectural disorganization was observed in these groups (Fig. 9). In the 90% oxygen groups, inflammatory cell infiltration was recognized after 72 hours of exposure, and alveolar septal destruction was apparent by 96 hours (Fig. 10). Diffuse alveolar damage was apparent after 120 hours of exposure.

## Discussion

In the present study, we have demonstrated that pulmonary epithelial functions, detected by sensitive markers were damaged and that both breakdown of collagen fibrils and fibrogenesis were transiently (in the 2 and 4 week-exposure groups) induced even with low-dose (40%) oxygen exposure. These changes were, however, successfully compensated by 8 weeks with continuous oxygen exposure, and neither significant epithelial injury nor fibrosis occurred by long-term low-dose (up to 40%) oxygen supplementation. On the other hand, 90% oxygen exposure caused rapid and progressive destruction of the lung without compensation. The clearance of Tc-DTPA was increased by 24 hours, inflammatory cell infiltration was recognized by 72 hours, and alveolar septal destruction by 96 hours and diffuse alveolar damage, as is observed in ARDS, by 120 hours in 90% oxygen exposure. Although neutrophils in BAL fluids were progressively increased after 72 hour-exposure to 90% oxygen, there were no significant changes in percentage of individual cell types in the 40% oxygen exposure group. The difference between the 40% and 90% oxygen exposure groups indicates that there is a critical oxygen concentration at which progressive lung injury is induced.

Collagen metabolism was sensitive even to low-dose oxygen exposure. Biochemical measurement of total lung hydroxyproline is a convenient method to quantitate lung collagen. Although hydroxyproline in the lung is found mostly in collagen, it must be noted that hydroxyproline is also found in another protein, i.e., in elastin [25,26]. Therefore, it has generally been recommended to conduct hydroxyproline measurement together with histological analysis to evaluate the fibrosing state of the lung [26]. In histological quantification of fibrous tissue and histopathological observation, it is thought that oxygen-exposed lungs show alveolar enlargement that can obscure fibrotic change even if the total fibrous tissue content increases [27]. This could explain why the results of our hydroxyproline measurement and histological analysis of fibrous tissue showed some discrepancy. Therefore, we conducted both hydroxyproline measurement and histological analysis. However, it should be noted that there might be the limitation of the histological quantification of fibrous tissue, although we obtained roughly matching result.

Sequential pathological and BAL fluid changes have shown that the breakdown of defense mechanisms against hyperoxia is closely related to inflammatory cell infiltration. It has been widely recognized that hyperoxia exerts its toxic effect by increasing oxygen radicals [28-30]. Contribution of excess reactive oxygen species released by hyperoxia-induced activated neutrophils has been pointed out [31,32], and blockade of the neutrophil influx has been shown to ameliorate lung injury in newborn rats [33]. Newborn rats exposed to 60% oxygen for 14 days developed a heterogeneous parenchymal lung injury with areas of arrested alveolarization, while suppression of neutrophil influx resulted in enhanced alveolar formation: airspace size variance was reduced, mean linear intercept was decreased, and secondary crest formation was enhanced [33]. These reports indicate that various reactive oxygen species generated from a respiratory burst of accumulated and activated neutrophils amplify the process of toxic oxygen-induced lung injury [34].

Hyperoxic lethal lung injury in the present study closely resembled endotoxin-induced acute respiratory distress syndrome (ARDS), suggesting that common mechanisms act on the lung injury. Firstly, histopathological findings observed in the 90% oxygen 120 hour-exposure group is substantially the same as those in ARDS; i.e. diffuse alveolar damage. Secondly, it has been reported that activated neutrophils play a central role in the development of lung injury in ARDS [35]. Finally, pulmonary endothelium is the target tissue in oxygen-induced lung injury and ARDS, both of which induce pulmonary edema and neutrophil infiltration into alveolar spaces. Hydrogen peroxide is produced by adherent granulocytes in the intact rat lung treated with endotoxin, which causes the granulocyte adhesion and oxidative stress to the endothelium within 30 min in the pulmonary microcirculation [36]. Although pulmonary inflammation is a CD18-independent event in a model of oxygen toxicity in the guinea pig [37], ICAM-1, the ligand of CD18, is indispensable for neutrophil H_2_O_2 _production in the pulmonary microcirculation of endotoxin-infused rats [38]. Upregulation of ICAM-1 has been demonstrated in hyperoxia-exposed pulmonary arterial endothelial cells *in vitro *[39,40]. Taken together, the mechanism that leads to the fatal lung injury by hyperoxic exposure might be induction of respiratory burst of activated neutrophils and subsequent endothelial injury such as by endotoxin. If a certain oxygen concentration is below the threshold needed to induce respiratory burst, the lung might be able to compensate for oxygen toxicity.

Imbalance between collagenolytic and collagenosynthetic activities has been hypothesized to induce pulmonary emphysema [41] and fibrosis [42]. Overexpression of interstitial collagenase causes pulmonary emphysema in mice [41], while low collagenase activity has been demonstrated in pulmonary fibroblasts obtained from patients with idiopathic pulmonary fibrosis [42]. In the present study, normal guinea pig lungs exposed to relatively low-dose oxygen concentration showed dynamic changes in collagenase activities which resulted in the collagen balance being maintained. This finding, observed in successful hyperoxia-induced remodeling in normal lungs, indicates a striking difference from that in disease states such as pulmonary emphysema and fibrosis.

## Conclusion

We conclude that epithelial functions are transiently damaged at 2 to 4 weeks and that both breakdown of collagen fibrils and fibrogenesis are induced even with low-dose (40%) oxygen exposure in the guinea pig lung. However, these changes are compensated by 8 weeks even with continuous oxygen exposure, and neither significant epithelial injury nor fibrosis occurs with long-term low-dose oxygen supplementation.

## Competing interests

The authors declare that they have no competing interests.

## Authors' contributions

TA designed the study, conducted most of the experiments and prepared the draft of the manuscript. FY conducted most of the experiments and data analysis. TK supervised the experiments and conducted most of the experiments. TS conducted experiments of collagen metabolism. AI supervised the experiments and conducted the animal experiments especially for the assay of inhaled Tc-DTPA clearance. TU gave helpful advices in the preparation of the manuscript. YO supervised the analysis of the experimental data and the preparation of the manuscript. All authors read and approved the final manuscript.
